# Research progress on testicular torsion secondary to inguinal cryptorchidism

**DOI:** 10.3389/fped.2026.1833244

**Published:** 2026-06-25

**Authors:** Qingyu Xu, Lin Zhang, Nan Cong, Yi Song, Qi Shi

**Affiliations:** 1 Department of Pediatric Surgery, Hong Qi Hospital Affiliated to Mudanjiang Medical University, Mudanjiang, China; 2 First School of Clinical Medicine, Mudanjiang Medical University, Mudanjiang, China; 3 School of Nursing, Mudanjiang Medical University, Mudanjiang, China; 4 Department of Pathology, Mudanjiang Tumor Hospital, Mudanjiang, China; 5 Graduate School of Mudanjiang Medical University, Mudanjiang, China

**Keywords:** clinical diagnosis, disease management, inguinal cryptorchidism, orchiopexy, testicular torsion

## Abstract

**Objective:**

Inguinal cryptorchidism, a common congenital anomaly in pediatric patients, is a major risk factor for testicular torsion. Its abnormal anatomical location and atypical clinical manifestations frequently lead to delayed diagnosis and misdiagnosis. This study aims to systematically review existing evidence to clarify the epidemiology, pathophysiology, diagnostic and therapeutic key points, and prognosis of testicular torsion secondary to inguinal cryptorchidism, with the goal of providing robust, evidence-based references for clinical practice.

**Method:**

This narrative review synthesizes and summarizes recent clinical studies, case reports, and related discussions on testicular torsion secondary to inguinal cryptorchidism, comprehensively analyzing current research findings on this condition.

**Result:**

Children with inguinal cryptorchidism have approximately a 10-fold higher risk of testicular torsion compared with those with normally descended testes in the scrotum. Typical clinical manifestations include an acute painful inguinal mass and an empty ipsilateral scrotum, which can easily be confused with incarcerated indirect inguinal hernia and other acute groin disorders. Missed diagnosis is a leading cause of increased testicular necrosis and orchiectomy rates. Color Doppler ultrasound is the preferred first-line imaging modality. The core therapeutic principle adheres to “Time is testis,” and urgent surgical exploration is indicated in cases with high clinical suspicion. Intraoperatively, testicular repositioning and fixation or orchiectomy are performed according to the evaluation of testicular viability. Prophylactic contralateral orchiopexy is strongly recommended. Long-term management mainly focuses on monitoring for testicular atrophy, reproductive function, and the risk of malignant transformation.

**Conclusion:**

The core principles for diagnosing and treating testicular torsion secondary to inguinal cryptorchidism are early recognition, imaging confirmation, emergent surgery, and long-term follow-up. Clinicians should maintain high vigilance for acute inguinal masses in infants and young children and regard an ipsilateral empty scrotum as a key differential diagnostic clue. Surgical delay caused by atypical symptoms must be avoided. Enhanced routine screening, optimized emergency assessment protocols, and standardized long-term follow-up can effectively improve the prognosis of affected children.

## 1 Introduction

**Figure 1:**
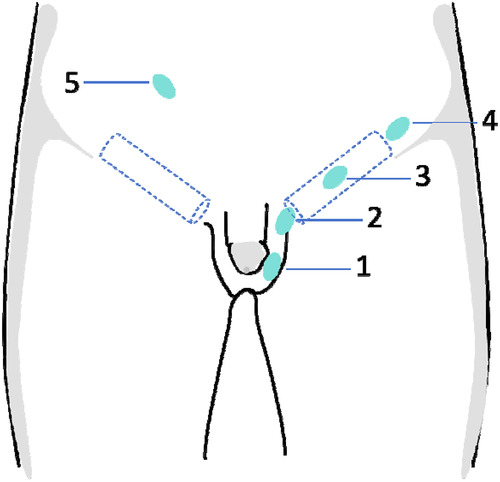
Schematic diagram of different testicular descent positions (1: normal intrascrotal position; 2: suprascrotal position; 3: inguinal canal; 4: internal inguinal ring; and 5: abdominal cavity) Figure created with PowerPoint Presentation.

Cryptorchidism is a congenital disorder characterized by the failure of the testes to descend along their normal physiological pathway into the scrotum. Instead, they remain in the abdominal cavity, inguinal canal, or other abnormal locations (1, 2). It is one of the most common congenital urogenital malformations in male infants. According to the retention site of the testis, cryptorchidism is classified into intra-abdominal, inguinal, and suprascrotal subtypes (3–5) (see Figure 1). Among these subtypes, inguinal cryptorchidism accounts for the majority of clinical cases (6). The inguinal cryptorchidism discussed in this review specifically refers to palpable undescended testes located within the inguinal canal, internal inguinal ring, or external inguinal ring, excluding ectopic testes. The anatomical features of each subtype are illustrated in Figure 1 to facilitate rapid clinical identification. Previous clinical studies have mainly focused on the associations between cryptorchidism and spermatogenic dysfunction, testicular malignancy, and concurrent inguinal hernia (7). However, its correlation with testicular torsion (TT) has received relatively limited attention. Testicular torsion is a surgical emergency caused by spermatic cord rotation, which results in impaired testicular blood perfusion. Without timely intervention, testicular ischemic necrosis will occur within a short period. The high-risk age groups for TT are infancy and adolescence (8). Clinical data demonstrate that patients with cryptorchidism have approximately 10 times the risk of testicular torsion compared with those with normal testicular position (9). Due to the special anatomical location of inguinal cryptorchidism, its clinical manifestations are non-specific, often misdiagnosed as incarcerated indirect inguinal hernia, acute epididymitis, inguinal lymphadenitis, or other conditions (10). Based on published clinical research and practical experience, this article reviews the epidemiological characteristics, pathogenesis, diagnosis and treatment strategies, and long-term prognosis of testicular torsion secondary to inguinal cryptorchidism, to provide a reference for clinical practice.

## 2 Epidemiological characteristics

Inguinal cryptorchidism is a well-established major risk factor for testicular torsion. Currently, most epidemiological studies focus on cryptorchidism or testicular torsion as separate entities, and population-based incidence data specifically for testicular torsion secondary to inguinal cryptorchidism remain insufficient. Cryptorchidism is a common congenital malformation of the male reproductive system, occurring in approximately 1.0%–4.6% of full-term newborns and up to 45.0% of preterm infants (11, 12). The inguinal subtype predominates in clinical practice (6). Approximately 80% of undescended testes are palpable on physical examination; most cases are unilateral, with a slight right-sided predominance (13). The overall incidence of testicular torsion is approximately 3.8 per 100,000 population (14), with two distinct age peaks: infancy (<1 year old) and adolescence (12–18 years old) (15, 16). Children with inguinal cryptorchidism have a 10-fold higher risk of testicular torsion compared with children with normally descended intrascrotal testes (9), which is mainly attributed to abnormal anatomical structures including anomalous testicular mesentery attachment, patent processus vaginalis, and excessive mobility of the spermatic cord (17, 18).

Data on racial and geographical disparities in testicular torsion secondary to inguinal cryptorchidism are scarce, especially large-scale multicenter studies focusing on Asian and Chinese populations. The overall prevalence of cryptorchidism varies across ethnicities and regions; several reports indicate a higher incidence among Caucasian populations, but subtype-specific analyses of inguinal cryptorchidism and its torsion risk are still lacking (19, 20). This article summarizes clinical experience from Chinese medical institutions to supplement data for Asian populations. Further rigorous multi-regional and multi-ethnic epidemiological studies are required to clarify the impacts of geography, genetics, and environmental factors on disease risk. Although inguinal cryptorchidism-related testicular torsion accounts for a small proportion of all TT cases, it carries great clinical significance (21). Atypical clinical presentations, especially in preverbal infants, frequently lead to misdiagnosis and delayed treatment. Consequently, the rates of testicular ischemia, necrosis, and orchiectomy are markedly higher than those of primary intrascrotal testicular torsion (22). A thorough understanding of epidemiological features is crucial for early identification, risk stratification, and timely intervention.

## 3 Pathogenesis and clinical features of inguinal cryptorchidism

### 3.1 Physiological process of testicular descent

Testicular descent during fetal development is a highly coordinated biological process, which is divided into two main phases: the abdominal phase and the inguinoscrotal phase (23). This process is regulated by multiple factors including insulin-like peptide 3 (INSL3), androgens, gubernaculum development, changes in intra-abdominal pressure, and neuroendocrine signaling pathways (24, 25). Any disruption during this process can cause arrested testicular descent, resulting in testes retained within the abdominal cavity, inguinal canal, or near the external inguinal ring. Inguinal cryptorchidism is defined as testicular retention within the inguinal canal or adjacent to the internal/external inguinal rings. Some children present with a movable inguinal mass, while testicular palpation is difficult in others due to high testicular position or surrounding tissue compression (26).

### 3.2 Pathological basis of inguinal cryptorchidism

Studies by Qingsong et al. (27, 28) confirmed that long-term exposure of testes to a temperature higher than the intrascrotal environment leads to dysplasia of seminiferous tubules, impaired Sertoli cell function, and varying degrees of damage to germ cells. When inguinal cryptorchidism is complicated by a patent processus vaginalis, anomalous spermatic cord attachment, redundant testicular mesentery, or loose peritesticular soft tissue connections, testicular mobility increases significantly, thereby elevating the risk of testicular torsion (29, 30). In addition, testes retained in the inguinal canal are vulnerable to compression by the narrow canal, external mechanical stimulation, and local inflammation. These factors destabilize regional blood circulation. Once torsion occurs, ischemic injury progresses rapidly and exacerbates testicular damage.

### 3.3 Clinical manifestations of inguinal cryptorchidism

Most children with inguinal cryptorchidism present with a unilateral or bilateral hypoplastic scrotum and an absent testis within the scrotum. The volume of the affected scrotum is generally smaller than the contralateral side. Palpation of the inguinal region may reveal a round, smooth, moderately firm, and slightly mobile solid mass, which represents the retained testis (31). The size, location, and palpability of this mass vary individually. Increased intra-abdominal pressure (e.g., during crying) or postural changes can make the inguinal mass more prominent or cause distal testicular migration due to cremaster muscle contraction and pressure conduction (32). Affected children are usually asymptomatic, experiencing no local pain, erythema, or swelling in the quiescent stage. This condition is easily overlooked by parents and non-specialist clinicians, creating potential risks for acute testicular torsion (33). Many cases are detected incidentally during routine physical examinations or assessments for unrelated diseases.

## 4 Association between inguinal cryptorchidism and testicular torsion

### 4.1 Cryptorchidism as a major risk factor for testicular torsion

The pathological essence of testicular torsion is the rotation of the testis around the longitudinal axis of the spermatic cord, which first obstructs venous outflow and causes testicular congestion and edema, followed by compression of the spermatic artery. This progressive cascade leads to reduced arterial perfusion, testicular ischemia, and hypoxia. Without timely detorsion, irreversible testicular necrosis can develop rapidly, resulting in permanent loss of endocrine and reproductive function (8, 34, 35). Normally descended intrascrotal testes are fixed by stable surrounding anatomical structures. In contrast, undescended testes have abnormal positioning, disordered spermatic cord attachment, and excessive mobility, leading to a substantially higher torsion risk. Inguinal cryptorchidism is a high-risk subgroup within this disease spectrum (36).

### 4.2 Clinical characteristics of testicular torsion complicating inguinal cryptorchidism

TT secondary to inguinal cryptorchidism predominantly occurs in infants and young children. Many patients have a history of a reducible inguinal mass that went unrecognized by caregivers; in other cases, cryptorchidism is first diagnosed at the onset of torsion. Compared with primary intrascrotal TT, inguinal cryptorchidism-related torsion has the following clinical features: (1) acute painful inguinal mass with obvious tenderness; (2) absent testis in the ipsilateral scrotum (37); (3) local skin erythema, swelling, and severe tenderness at the lesion site; (4) infants cannot verbalize discomfort and present with non-specific symptoms such as unexplained crying, irritability, and poor oral intake, occasionally accompanied by fever and vomiting; and (5) with prolonged ischemia, the inguinal mass becomes tense, local skin temperature rises, and systemic inflammatory responses, including fever and leukocytosis, develop. Due to the absence of typical scrotal signs, this condition is frequently misdiagnosed as incarcerated hernia, inguinal lymphadenitis, testicular appendage torsion, or superficial soft tissue infection (38).

### 4.3 Torsion angle and necrosis risk

The severity of testicular injury is closely correlated with the torsion angle and ischemic duration (39) (Table 1). In general, larger torsion angles cause more complete vascular occlusion, which accelerates the progression of necrosis. Clinically, extreme torsion exceeding 720° or even 1,800° can be observed, which leads to near-complete strangulation of testicular vessels. The affected testis appears dark, dull, and non-elastic; perfusion is rarely restored even after detorsion, necessitating orchiectomy. The insidious presentation of torsion in infants with inguinal cryptorchidism often results in diagnosis beyond the optimal treatment window, significantly increasing the risk of testicular necrosis.

**Table 1:** Comparison chart of testicle torsion degree and necrosis time.

Torsion degree	Estimated necrosis time	Clinical features and risk descriptions	References
90°	Approximately 7 days	Partial blood flow obstruction; relatively lower necrosis risk but still requires timely intervention. Individual tolerance varies considerably.	(40)
180°	3–4 days	Marked venous obstruction; testicular swelling develops. Surgical detorsion is required within 3 days.	(41)
360°	12–24 h	Complete arterial occlusion; severe testicular ischemia. This is the most common clinical presentation.	(42)
540°	6–12 h	Complete vascular compression; salvage rate is extremely low even with intervention within 6 h.	(43)
≥720°	2–4 h	Critically severe ischemia; infarction can occur within 2 h. Significant individual variability exists.	(44–46)

The ischemic tolerance window is a population-based reference range and not an absolute criterion. Testicular survival is also influenced by age, spermatic cord anatomy, and collateral circulation capacity. Final decisions should be based on intraoperative assessment of testicular vitality.

## 5 Clinical diagnosis of testicular torsion secondary to inguinal cryptorchidism

### 5.1 Medical history collection

For infants and young children presenting with acute painful inguinal swelling, key historical information includes (1) whether the ipsilateral scrotum is hypoplastic or devoid of a testis; (2) whether previous physical examinations confirmed an undescended testis; (3) the onset time of the inguinal mass, whether it developed acutely, and whether symptoms are aggravated during crying (31); (4) presence of accompanying systemic symptoms such as fever, vomiting, poor oral intake, or lethargy; (5) whether the inguinal mass was previously reducible; and (6) history of inguinal hernia or similar symptomatic episodes. A combination of an ipsilateral empty scrotum and an acute inguinal mass strongly suggests testicular torsion in an undescended inguinal testis.

### 5.2 Physical examination

Physical examination is a crucial step for early diagnosis. Key examination items include (1) symmetry and developmental status of bilateral scrota; (2) presence or absence of a testis in the affected scrotum (37); (3) presence of a fixed, tender mass in the inguinal region (31); (4) consistency and tension of the mass; (5) local skin erythema, swelling, or increased skin temperature; and (6) position and morphology of the contralateral testis. Infants should be examined gently in a calm state; observation of mass changes during crying is feasible when necessary (32). Physical findings of an empty ipsilateral scrotum combined with a palpable, tender, and irreducible inguinal mass establish testicular torsion as the primary differential diagnosis.

### 5.3 Color Doppler ultrasound

Color Doppler ultrasound is currently the most widely used and convenient first-line imaging modality for this condition. Its diagnostic value includes (1) confirming the exact anatomical location of the testis and establishing the diagnosis of cryptorchidism; (2) evaluating testicular size, morphology, and internal echotexture to identify testicular swelling and abnormal echoes (9); (3) assessing the integrity of the tunica albuginea and the presence of peritesticular effusion; (4) detecting blood flow signals within the testicular parenchyma and spermatic cord and measuring arterial resistive index (RI) and pulsatility index (PI); (5) identifying characteristic torsion signs such as the whirlpool sign (47, 48); and (6) differentiating this condition from incarcerated hernia, lymphadenitis, and other inguinal masses. Typical ultrasound manifestations include thickened and hyperechoic inguinal subcutaneous tissue, a hypoechoic or heterogeneous solid inguinal mass, testicular enlargement, decreased or disordered internal echoes, markedly reduced or absent intratesticular blood flow, and visible spermatic cord torsion in some cases (49). With prolonged ischemia, the internal echotexture changes from homogeneous to heterogeneous, indicating hemorrhagic infarction or necrosis (50).

### 5.4 Other auxiliary examinations

Routine blood tests and C-reactive protein levels can indicate systemic inflammatory responses but lack diagnostic specificity for testicular torsion, serving only as adjunctive evidence. CT and MRI are not recommended for initial emergency diagnosis; they are reserved for clinically stable patients facing diagnostic dilemmas or requiring further delineation of local anatomical relationships (51). In cases with high clinical suspicion of testicular torsion, surgical intervention should not be delayed while waiting for additional imaging results.

## 6 Differential diagnosis of testicular torsion secondary to inguinal cryptorchidism

The non-specific symptoms of this condition are easily confused with other acute inguinoscrotal disorders. Accurate differential diagnosis is crucial to avoid treatment delays and improve the testicular salvage rate. When a child with unilateral cryptorchidism presents with acute inguinal pain, torsion of the ipsilateral undescended testis must be prioritized in the differential diagnosis. For rare cases of bilateral cryptorchidism, unilateral torsion warrants close monitoring for contralateral torsion due to shared underlying anatomical abnormalities and disease risks. During emergency management of the affected side, physical examination and ultrasound should be used to evaluate the position, morphology, and blood flow of the contralateral testis. Concurrent bilateral torsion should be suspected if contralateral pain, swelling, or abnormal blood flow is detected, necessitating bilateral surgical exploration intraoperatively. Therefore, confirming bilateral cryptorchidism during history taking and physical examination is key to avoiding missed contralateral lesions. The common characteristics of differential diagnoses are shown in Table 2.

**Table 2:** Differential diagnosis of inguinal undescended testis with testicular torsion.

Diagnosis of diseases	Clinical features	Signs and history	Color Doppler ultrasound findings	Core differential points	References
Incarcerated indirect inguinal hernia	Sudden painful inguinal mass; crying.	Often reducible; associated with intestinal obstruction (vomiting, distension, obstipation); ipsilateral scrotal testis palpable.	Bowel loops/contents or anechoic fluid; bowel wall blood flow; normal scrotal testis with preserved flow.	Incarcerated hernia: normal ipsilateral scrotal testis; ultrasound shows bowel contents rather than torsed testicular tissue.	(37)
Inguinal lymphadenitis	Painful inguinal mass; possible fever.	Recent infection; multiple mobile enlarged lymph nodes; normal ipsilateral testis.	Discrete oval/round hypoechoic nodules with clear margins and hilar/peripheral flow; normal testicular structure and flow.	Lymphadenitis: nodal architecture, clear infection history, normal ipsilateral testis.	(52)
Testicular appendage torsion	Scrotal/inguinal pain; milder than torsion.	Normal scrotal testis; localized tenderness.	Normal or slightly increased testicular flow; small avascular nodule near testis/epididymis; reactive hydrocele.	Almost exclusively affects normally descended scrotal testes; preserved testicular blood flow.	(53–55)
Acute epididymitis/orchitis	Groin/scrotal pain/swelling; fever.	Urinary tract infection or systemic infection; gradual onset; normal testicular position.	Enlarged epididymis/testis with heterogeneous echoes; markedly increased color flow; hydrocele; normal testicular location.	Normal scrotal testicular position with increased blood flow signals.	(56, 57)
Local cellulitis or abscess	Inguinal erythema, warmth, pain; possible fluctuant mass.	Prominent skin infection signs; high fever, leukocytosis; normal testis.	Diffuse subcutaneous edema and thickening; irregular anechoic abscess cavity; normal testicular structure and flow.	Infection involves superficial soft tissues rather than the testis itself.	(38, 49)

## 7 Treatment principles and clinical management

Urgent surgical exploration is mandatory for patients with a high clinical suspicion of testicular torsion secondary to inguinal cryptorchidism (58). Any observational waiting or repeated imaging examinations will increase the risk of testicular necrosis, especially in infants and young children. Even if ultrasound findings are atypical, active surgical intervention is indicated when history and physical examination strongly suggest torsion (59). Intraoperative evaluation focuses on (1) the number and direction of testicular rotation; (2) the congestion and ischemic status of spermatic cord vessels; (3) the color, tension, and elasticity of the testis; (4) the integrity of the tunica albuginea and surrounding anatomical structures; (5) blood flow recovery after testicular detorsion; and (6) anatomical abnormalities of the contralateral testis and indications for simultaneous fixation (60).

**Figure 2:**
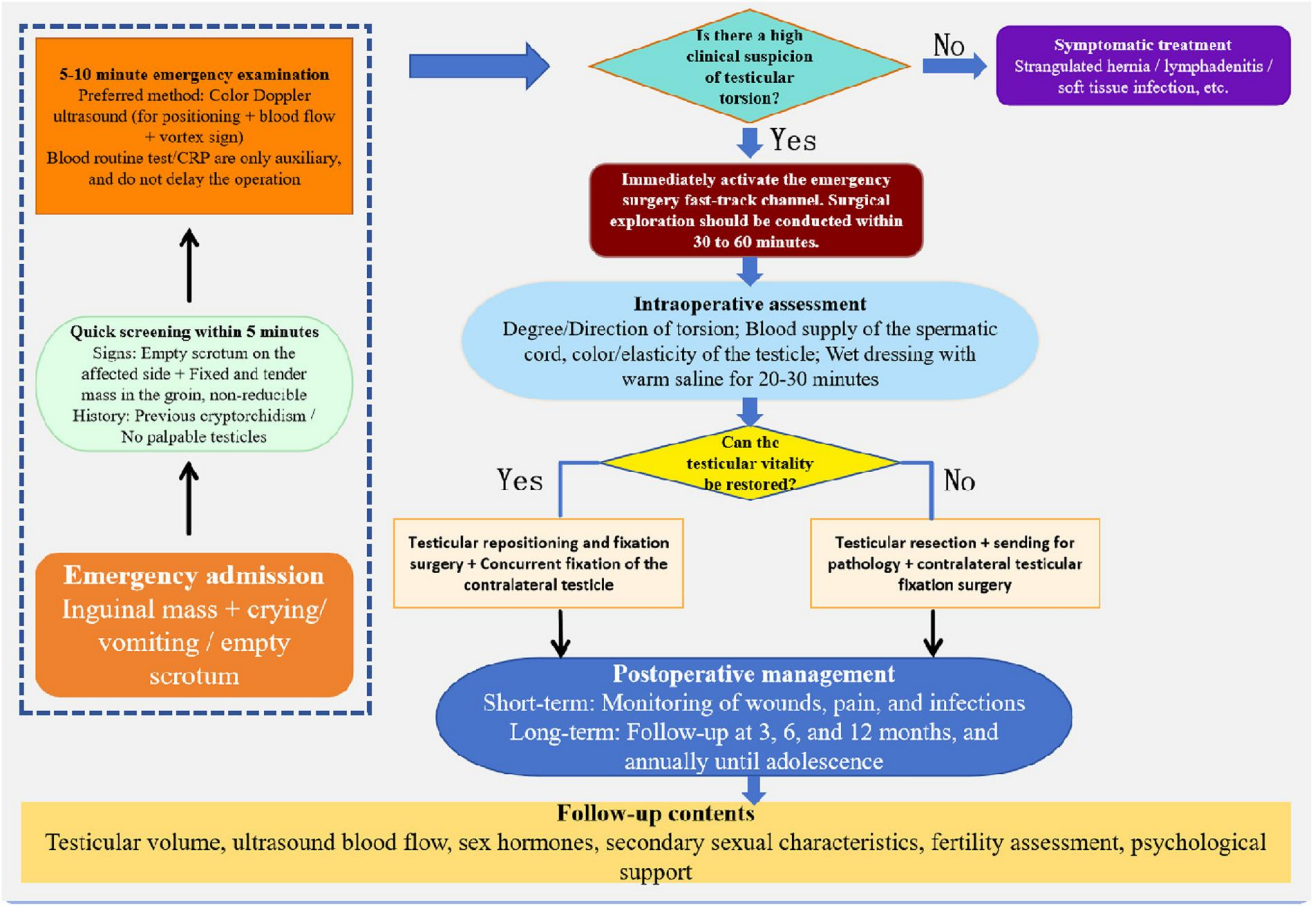
Clinical diagnosis and treatment flowchart for testicular torsion secondary to inguinal cryptorchidism. Drawn from PowerPoint Presentation.

Intraoperative assessment of testicular viability is crucial for deciding between testicular preservation and orchiectomy (61): (1) Orchiectomy: This is indicated when the testis exhibits dark purple discoloration, complete loss of luster and elasticity, absence of fresh bleeding after tunica albuginea incision, and failure to restore perfusion after 20–30 min of warm saline gauze wrapping. (2) Orchiopexy: This is performed when the testis shows improved color and texture with capillary bleeding after detorsion, indicating reversible perfusion. The testis is then surgically preserved and fixed (62). Standard testicular descent and fixation surgery is the key procedure to prevent recurrent torsion in viable torsed testes or for the elective management of inguinal cryptorchidism. The inguinal approach is most commonly used as allows for full dissection of the spermatic cord, closure of a patent processus vaginalis, and repair of concurrent indirect inguinal hernia, making it the standard surgical method for inguinal cryptorchidism (63). The classic tunica vaginalis fixation technique involves creating a subdartos pouch to secure the testis at the scrotal base, preventing retraction and recurrent torsion (22). Laparoscopic-assisted transscrotal orchiopexy is increasingly applied for palpable inguinal cryptorchidism, offering potential advantages in operative time, postoperative recovery, and cosmetic outcome (64). Regardless of the surgical approach, the primary goal is to achieve tension-free fixation with preserved blood flow and secure placement at the scrotal base. Due to potential risks such as bilateral testicular anatomical abnormalities or excessive testicular movement in some children, contralateral prophylactic orchiopexy is routinely recommended (61), especially for children with only one functional testis. Irreversibly necrotic testes should be resected decisively. Forced retention not only fails to restore function but can also cause sterile necrosis absorption, secondary inflammation, and parental misunderstanding of treatment outcomes (65). Resected testicular specimens should undergo routine pathological examination to confirm diagnosis and rule out rare malignant lesions (66). Baldanza et al. (14) reported a case in which the patient presented more than 24 h after symptom onset; delayed diagnosis and treatment resulted in irreversible testicular necrosis and loss of salvage opportunity. A standardized long-term postoperative follow-up protocol should be established for all patients (Figure 2).

## 8 Prognosis and long-term management

Treatment outcomes for TT are closely related to the time from symptom onset to medical intervention (67). In general, earlier detorsion is associated with higher testicular salvage rate; as ischemic time increases, testicular damage worsens and salvage rates decline sharply (68). For scrotal TT, existing data indicate that salvage rates exceed 90% if detorsion surgery is completed within 6 h of symptom onset, but fall below 10% when ischemia exceeds 24 h (41). However, the diagnosis of inguinal cryptorchidism torsion is often delayed due to its concealed anatomical location and atypical clinical manifestations. Consequently, the overall testicular salvage rate is significantly lower than that for testicular torsion within the scrotum. Even if a testicle is successfully preserved, postoperative testicular atrophy is a long-term complication that requires attention. Studies show that a considerable proportion of successfully repositioned testicular torsions will experience varying degrees of testicular volume atrophy (69). Therefore, regular postoperative ultrasound monitoring of testicular volume and internal echotexture is critical for evaluating long-term function.

All children with inguinal cryptorchidism, regardless of whether torsion occurs, require long-term management that includes monitoring for malignancy risk. Cryptorchidism is a clear risk factor for testicular germ cell tumors; even after testicular descent fixation surgery, the risk remains higher than that of the normal population (70, 71). After puberty, patients should be enrolled in a long-term testicular tumor monitoring program. Key measures include educating patients on monthly testicular self-examination and regular urological follow-up into adulthood. For children with high-position inguinal cryptorchidism or those with high-risk factors such as gonadal dysplasia, regular testicular examination may be considered. Any finding of painless swelling, hard texture, or nodules of the testicle should prompt immediate medical consultation for further examination.

Following unilateral orchiectomy, most children with a normal contralateral testis maintain basic endocrine and reproductive function. However, the contralateral testis also requires long-term follow-up because cryptorchidism is associated with abnormal germ cell development. While the psychological impact of unilateral testicular absence is not obvious during childhood, as the child grows older, the child and their family may experience anxiety due to appearance, sexual development, and future marriage and childbearing issues (72). Therefore, adequate health education and psychological support should be provided during postoperative follow-up. For patients with clear needs, testicular prosthesis implantation may be considered at an appropriate age to improve body image and relieve psychological distress.

## 9 Standardized management strategies for inguinal cryptorchidism and testicular torsion

### 9.1 Strengthen routine physical examination and screening for newborns and infants

Systematic examination of the reproductive system should be integrated into routine physical examinations for newborns, infants, and children during wellness visits and vaccinations (73). This examination should assess testicular position, size, texture, and symmetry. If testicles are not palpable or have not fully descended into the scrotum, their specific retention location (e.g., within the internal or external rings of the inguinal canal, at the entrance of the scrotum) should be clearly recorded, and a graded management mechanism should be initiated (74). Primary care providers, pediatricians, and community clinicians serve as frontline gatekeepers for early cryptorchidism detection and require specialized training to enhance recognition of cryptorchidism and its potential complications (torsion, malignancy), ensuring timely referral to pediatric surgery or urology for professional assessment (75). A study by Keiichiro et al. (76) confirmed that standardized screening programs can effectively improve the early detection rate of cryptorchidism. In addition, an electronic medical record tracking system should be established for long-term follow-up of affected children.

### 9.2 Standardize the timing of elective surgery for cryptorchidism

It is widely recognized in clinical practice that cryptorchidism should be evaluated and treated with elective fixation surgery at an appropriate age to maximize the preservation of spermatogenic function and reduce the long-term risks of torsion and malignancy. For palpable inguinal cryptorchidism, elective testicular descent fixation surgery is the standard treatment method. The recommended surgical timing is 6–12 months of age, and no later than 18 months (77). Clinicians should proactively educate parents on the necessity of early surgery and clearly explain the risks of delayed intervention, including subfertility and TT (78). Preoperative color Doppler ultrasound should be performed to define testicular location, size, and associated anomalies such as patent processus vaginalis or inguinal hernia, providing a basis for precise surgical planning (79).

### 9.3 Improve awareness of emergency differential diagnosis

For infants presenting with an “acute inguinal mass accompanied by pain or crying,” emergency department, pediatric, and radiology personnel need to include TT secondary to inguinal cryptorchidism as one of the primary differential diagnoses. Clinical decision-making should closely adhere to the core clue of an “ipsilateral empty scrotum.” An emergency assessment fast-track should be established, integrating history inquiry, key physical examination, and urgent color Doppler ultrasound examination. The ultrasound examination request form should clearly indicate “testicular torsion” as the clinical suspicion, prompting the ultrasound physician to specifically search for key signs such as “reduced or absent testicular parenchymal blood flow” and “testicular cord vortex sign” (47, 48). For high clinical suspicion of torsion, treatment should not be delayed to await laboratory results or non-specific ultrasound findings. Strict adherence to the time is testis principle is mandatory, with prompt decision-making for surgical exploration.

### 9.4 Establish a standardized long-term postoperative follow-up system

Surgery marks the beginning of long-term management. A multi-dimensional postoperative follow-up system should be established: (1) Regular follow-up should occur at 3, 6, and 12 months postoperatively, then annually until puberty, focusing on clinical and ultrasound assessment of testicular position, size, and consistency (49). A long-term follow-up study by Uijldert et al. (78) confirmed that comprehensive follow-up data are crucial for accurately assessing surgical complications and long-term testicular development. (2) After the child enters puberty, the follow-up content expands to include the development of secondary sexual characteristics (80). For children with unilateral testis removal or suspected bilateral testis dysfunction, appropriate tests for serum testosterone, follicle-stimulating hormone, luteinizing hormone, etc. should be conducted to assess endocrine function (81). (3) Semen analysis and professional reproductive counseling should be provided in early adulthood or before marriage (82). (4) Psychological support for patients and families is essential. For children who experience anxiety due to unilateral testis loss, relevant information and options for testis prosthesis implantation should be provided at an appropriate time to improve appearance and relieve psychological stress (72). Throughout the follow-up process, adequate health education should be conducted to ensure that family members clearly understand the goals and significance of long-term disease management (83).

## 10 Conclusions and prospects

Inguinal cryptorchidism complicated by TT is a difficult-to-diagnose pediatric urological emergency in clinical practice. Although it accounts for a relatively small proportion of all TT cases, its abnormal anatomical location, lack of specific symptoms, and the inability of young children to clearly express their discomfort, it is easily confused with incarcerated hernia, lymphadenitis, and other diseases. The high rate of clinical misdiagnosis and missed diagnosis often leads to testicular ischemic necrosis and an increased resection rate. Therefore, this critical and urgent condition requires high vigilance.

This review systematically summarizes the epidemiology, pathophysiology, clinical features, and treatment strategies for this condition, highlighting four core treatment principles: First, strengthen early identification: Clinicians must be vigilant for acute, painful masses in the groin area of infants and young children. “Ipsilateral empty scrotum” should be considered a key differentiating factor. A thorough history of cryptorchidism and physical examinations are crucial. Second, rely on ultrasound for precise diagnosis: Color Doppler ultrasound is the preferred auxiliary examination. The position, shape, and blood flow changes of the testicles should be comprehensively evaluated based on the medical history and physical examination. Third, adhere to the principle of “time is of the essence for testicles”: For highly suspected cases, immediate surgical exploration should be performed without awaiting for of auxiliary examination results. During the operation, the vitality of the testicles should be evaluated to decide between repositioning and fixation, or resection. At the same time, it is recommended to perform prophylactic contralateral orchiopexy to prevent the risk of torsion on the other. Fourth, implement long-term systematic management: This includes early surgical intervention for cryptorchidism to reduce torsion risk, as well as lifelong follow-up after surgery to monitor testicular development, endocrine function, reproductive potential, and psychological wellbeing.

To enhance diagnostic and treatment capabilities for children and improve their prognosis, future clinical work should refine the relevant research system, establish a multicenter case registration platform, and improve standardized data collection norms. Concurrently, prospective studies can explore predictive indicators affecting testicular preservation rates and verify the application value of new technologies such as contrast-enhanced ultrasound and shear wave elastography in disease diagnosis and long-term assessment.

This condition requires clinicians to possess acute awareness, diagnostic ability, and long-term management concepts. By implementing the research directions outlined above and promoting standardized systems, it is expected to further improve evidence-based treatment pathways and effectively improve the clinical prognosis and long-term quality of life for children.
